# High-risk pregnant women’s experiences of the receiving prenatal care in COVID-19 pandemic: a qualitative study

**DOI:** 10.1186/s12884-022-04676-1

**Published:** 2022-04-26

**Authors:** Kobra Mirzakhani, Nahid Jahani Shoorab, Azam Akbari, Talat Khadivzadeh

**Affiliations:** 1 Nursing and Midwifery Care Research Center, Mashhad University of Medical Sciences, Mashhad, Iran; 2 Department of Midwifery, School of Nursing and Midwifery, Mashhad University of Medical Sciences, Mashhad, Iran; 3 Emam Reza Hospital, Mashhad University of Medical Sciences, Mashhad, Iran

**Keywords:** Pregnancy, High risk, Pandemic, COVID-19, Prenatal care

## Abstract

**Background:**

Women with high-risk pregnancies are among the most vulnerable groups that require additional precautionary measures against the spread of COVID-19 plus receiving prenatal care. Yet, there is limited information on the status of prenatal care in women with high-risk pregnancies. The purpose of this study was to explore the experiences of women with high-risk pregnancies who were receiving prenatal care during the COVID-19 pandemic.

**Methods:**

The present qualitative study was conducted on mothers with high-risk pregnancies from September 2020 to March 2021. Purposeful sampling continued until achieving data saturation. Ghaem, Ommolbanin, and Imam Reza in Mashhad, Iran served as the research environment. Face-to-face and semi-structured interviews were effective data collection methods. Each interview lasted between 20 to 45 min (on average 30). The total number of participants was 31. Data analysis was carried out simultaneously with data collection using the qualitative content analysis method developed by Granheim and Landman (2004).

**Results:**

Following the reduction and analysis of data from women in high-risk pregnancies, as well as their perceptions and experiences with health services during the COVID-19 pandemic, eight subcategories and three main categories were identified, including 1) “Negative psychology responses,” 2) “Adoption behavior,” and 3) “Adjustment of health services in mutual protection.”

Fear, anxiety, stress, feelings of loneliness, sadness, depression, guilt, doubt and conflict in receiving services were examples of negative psychological responses. The adaptive behaviors’ category reflected the behaviors of women with high-risk pregnancies in the context of the COVID COVID-19 pandemic. The Adjustment of health services in mutual protection indicated that health workers took preventive and protective measures against COVID-19, which, in addition to protecting themselves and their clients against COVID-19, gave women a sense of security.

**Conclusion:**

Receiving prenatal care during the COVID-19 pandemic presents challenges for women with high-risk pregnancies, negatively impacting their psychological state and health-seeking behavior. Supportive and preventive care can ensure that women with high-risk pregnancies receive optimal prenatal care that focuses on COVID-19 prevention. We recommend implementing screening, psychological counseling, and education for women with high-risk pregnancies, as well as ensuring that they have access to women-centered health care services.

## Background

The outbreak of coronavirus is a global health threat [1]. Following the global pandemic crisis, the diagnosis of two coronavirus cases in Iran, the city of Qom, was confirmed on February 2020, and the outbreak of the new coronavirus was officially declared on March 2020, covering almost all provinces of the country [2].

COVID-19 is a beta-coronavirus that can be transmitted through physical contact between humans. According to studies, a person infected with COVID-19 can infect at least four other people with a new infection [3]. Overall, the hospital admission rate for this disease is 23% and the resulting death rate is 1–2% [4]. People with underlying diseases have been reported to have higher morbidity and mortality rate [5].

Pregnant women are one of the most vulnerable groups that require additional precautions against the COVID-19 outbreak [6]. Changes during pregnancy may increase susceptibility to some intracellular pathogens, particularly viruses, intracellular bacteria, and parasites [7]. Furthermore, high-risk pregnancies occur when the pregnant mother has underlying problems before or during pregnancy, in which case her physical, psychological, and social vulnerability increases [8]. The prevalence of high-risk pregnancies in Iran and other countries ranges from 25.6 to 75.6% [9, 10]. High-risk pregnancies significantly weaken the mother’s immune system and increase the risk of COVID-19 infection [11].

COVID-19 in pregnancy has been associated with complications such as premature rupture of the membranes (PPROM) and preterm delivery [12, 13], fetal distress, and fetal growth restriction as a result of maternal hypoxia. The rate of cesarean section is also reported to be 96.4%, possibly indicating that iatrogenic reasons (fear of obstetrics) can also be a factor [14, 15]. This has resulted in an increase in the number of pregnant women admitted to the ICU during the COVID-19 pandemic [16]. Therefore, until further data is available, it is recommended that pregnant women, particularly those with high-risk pregnancies, be completely protected from infection. Furthermore, it is recommended that all pregnant women avoid high-risk areas such as hospitals and health centers and have limited physical contact with health care workers, in other words, maintain social and physical distance [5]. These guidelines are useful for disease prevention [14]. However, studies have shown that mothers with high-risk pregnancies are more stressed in these situations due to concerns about their own and the fetus’s health, which increases anxiety and depression in high-risk pregnancies [17]. On the other hand, in high-risk pregnancies, the woman may experience stress due to the lack of access to health services [18]. As a result, high-risk pregnancy conditions, social distancing while limiting the number of visits to the woman can increase the woman’s stress, anxiety, and worry. During the COVID − 19 outbreak, people may not think clearly or react logically due to high levels of fear and anxiety [19, 20]. Maternal psychological distress (such as stress, anxiety, and depression) has consequences for both mother and fetus, and has been found to be a risk factor in children and associated with adult neurodevelopmental disorders, such as attention deficit hyperactivity disorder (ADHD), autism spectrum disorder (ASD), schizophrenia spectrum disorders, antisocial behavior, and depressive symptoms [21]. As a result, it is necessary to cope with the psychological distress caused by COVID-19 in the presence of its prevalence to avoid the side effects of fetal growth and neurodevelopmental disorders [12, 13].

So far, few quantitative studies have been conducted on the psychological effects of coronavirus outbreaks in the general population [22–24] and pregnant women [15, 25], and recommendations for receiving remote prenatal care have reduced referrals for prenatal care [26]. However, no qualitative studies have been conducted to explain the perceptions and experiences of women with high-risk pregnancies receiving maternity care under these conditions. Since cultural and social contexts are not taken into account when conducting quantitative studies, hence individual experiences, human interactions, feelings, perceptions, and thoughts cannot be measured quantitatively [27, 28]. Women with high-risk pregnancies, on the other hand, are vulnerable populations with numerous health consequences and risks; thus, understanding their perspective on receiving care in the context of the Corona pandemic condition, as well as discovering their stresses and challenges, is critical for appropriate interventions. Given the scarcity of extensive research on the experiences of women with high-risk pregnancies, research on their experiences and understanding of receiving prenatal care, as well as identifying the support they require in the socio-cultural context of Iranian society, appears necessary. As a result, the aim of this study was to explore how women with high-risk pregnancies perceived and experienced COVID-19 pandemic care.

## Methods

### Study design

The purpose of this study was to explore the experiences of women with high-risk pregnancies in receiving prenatal care during the COVID-19 pandemic; thus, the current qualitative study explores and provides deeper insights into real-world problems [29]. Unlike quantitative research, which collects numerical data points and intervenes or introduces treatments, qualitative research collects participants’ experiences, perceptions, and behavior in various contexts [30]. The current study was conducted from September 2020 to March 2021.

### Setting and participant recruitment

The research setting comprised high-risk maternity wards and high-risk prenatal clinics at three government and educational hospitals in Mashhad, Iran (Ghaem, Imam Reza, and Ommolbanin). These environments were created to accommodate easy access and a maximum diversity of participants. The study population included all women with high-risk pregnancies based on Centers for Disease Control and Prevention (CDC) indicators [31]. Participants included eligible women who were willing to participate in the study and were able to communicate with the researcher to share their experiences. Purposeful sampling was performed to ensure maximum variation in terms of age, gestational age, socioeconomic status, and type of complication associated with pregnancy.

After referring to the participants, the researcher introduced herself and explained the purpose of the research, all while adhering to the ethical codes of qualitative studies. The confidentiality of the information was explained to the interviewee, and after obtaining their informed written consent, they were able to participate in the study. Furthermore, participants had the right to withdraw from the study at any time.

### Data collection

The data was gathered through semi-structured individual interviews. The interview took place in a hospital or clinic, in a quiet room with enough facilities to provide comfort to a high-risk pregnant mother. Sampling was continued until data saturation. Thus, following data saturation with 28 participants, three interviews were conducted to ensure data saturation. There was no data and no new category formed in the last three interviews. The total number of people who took part was 31. Every participant was interviewed once. The semi-structured interview focused on participants’ perceptions and experiences receiving prenatal care during the COVID-19 pandemic. Questions include the following:

1. What was your experience with prenatal care during the Corona pandemic?
2. How do you feel about being pregnant in this situation?
3. How do you feel when you apply for prenatal care?
4. How is your access to prenatal care in the face of the Corona pandemic?
5. Discuss the factors that make you feel good about receiving health services.
6. Discuss the factors that make you feel uneasy/bad about receiving health services.
7. How does the environment and location of health delivery affect health services receipt?
8. What limitations /restrictions did you face when seeking health services?

In-depth questions like “Can you describe more?” “Could you elaborate?” and “Could you give an example?” were used during the interview to clarify explanations, sum up and summarize the interview, and obtain feedback to ensure that the researcher understood correctly. In the end, the participant was asked one final question: is there any topic left unanswered that you want to talk about it? The interview lasted 20–45 min (on average 30 min) depending on the amount of information and the participants’ conditions. After obtaining permission from the participants, the interviews were entirely recorded on an MP3 player.

### Data analysis

Data analysis was carried out concurrently with data collection using the Granheim and Landman (2004) method of qualitative content analysis [32]. Thus, in the present study, interviews were considered as a unit of analysis. After each interview, the researcher (first author) would listen to the audio file several times to get an overall view of it. All of the interviews’ text was then converted to a verbatim transcription. Sentences or paragraphs related to the main concept were identified as meaning units. The meaning units were reviewed several times before being assigned appropriate codes. Following the reduction and compression process, similar codes were merged, and subcategories appeared. Thus, the declining trend in data reduction continued until the final category with a more general and abstract meaning were extracted. Data analysis and management were carried out using the MAXQDA software (v. 10, VERBI Software GmbH, Berlin).

### Trustworthiness

For the accuracy and trustworthiness of the data in the fieldwork, the Lincoln and Guba (1985) method was used, which included credibility, dependability, confirmability, and transferability [33]. To ensure the credibility of the data, the researcher thought about it constantly, even during daily activities (constant engagement with the data). The coded texts were made available to other members of the research team (second, third, and fourth authors) for discussion and validation of the extracted codes, subcategories, and categories (peer review). Three participants were also given codes extracted from the text and asked to confirm or revise the researcher’s correct understanding of their experiences (member check). To ensure data dependability, the transcripts of the audio files and interviews were provided to three qualitative analysts; data were then coded and analyzed separately, yielding similar results to the researcher’s analysis and coding. To achieve confirmability, all stages of the study, including audio files and interview transcripts, were meticulously documented from beginning to end, allowing the article to be audited by experts if deemed necessary. To express transferability, more information about the participants, including demographic information, age, education, number of pregnancies, type of pregnancy complication, and gestational age, were fully provided in the present study. Also, the principle of maximum variation was followed in the selection of participants.

## Results

### The participants

**Fig. 1:**
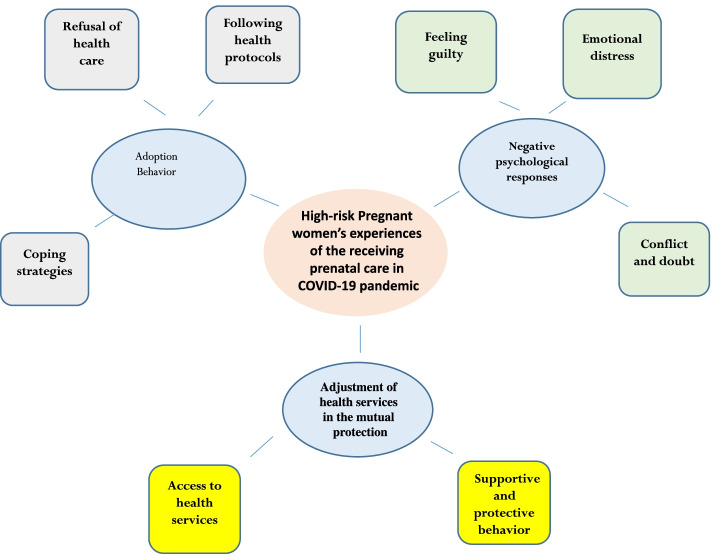
High-risk Pregnant women’s experiences of the receiving prenatal care in COVID-19 pandemic

Participants’ age ranged from 20 to 47 years old, with a mean of 30.34 ± 7.7 years. The gestational age ranged from 8 to 41 weeks. In terms of education, 10(31%) of the participants had primary and secondary education, 8(25.8%) had a diploma, and 13(41.93%) had a university education. The number of children among the participants ranged from none to four children. There were 16 women without children (51.61%) and 15 women with children (48.38%). Following data analysis and reduction, three categories including “Negative psychological responses,” “Adoption behavior,” and **“**Adjustment of health services in the mutual protection” were extracted (Table 1) (Fig. 1).

**Table 1:** The categories and subcategories which emerged from the analysis

Subcategories	Categories
**Emotional distress**	**Negative psychological responses**
**Conflict and doubt**	
**Feeling guilty**	
**Following health protocols**	**Adoption behavior**
**Refusal of health care**	
**Coping strategies**	
**Supportive and protective behavior**	**Adjustment of health services in the mutual protection**
**Access to health services**

#### 1-negative psychological responses

This category indicates that women in high-risk pregnancies experienced increased negative affects as a result of the COVID-19 pandemic, in addition to experiencing negative emotions associated with high-risk pregnancies. The Three subcategories of this main category were “Negative affect of high risk pregnant mothers during Covide’s conditions”, “Feelings of doubt and conflict in receiving health services”, “Feeling guilty about the complications of pregnancy during COVID-19 conditions”.

##### Emotional distress

When women in high-risk pregnancies sought to receive health care services during the COVID-19 pandemic, they experienced negative emotions such as fear, worry, stress, and anxiety about developing COVID-19. Because of the high-risk pregnancy conditions, they were terrified of developing COVID-19, which they imagined would put them and the fetus at greater risk of harm and danger. They were especially afraid about becoming infected with COVID-19 when they were admitted, hospitalized, or delivered in hospitals designed specifically for COVID-19 patients.

“Pregnancy during the Corona disease pandemic is filled with fear and stress, especially if the pregnancy is difficult. Our bodies weaken during pregnancy, and now that I also have gestational diabetes, getting infected with this Corona in the middle of the pregnancy would leave my child and I no chance of survival” (p 5).

They were also concerned about spreading the virus to other family members, particularly other children, from the hospitals and clinics or health centers to which they had referred to receive health services. Mothers with high-risk pregnancies who had another child at home, on the other hand, had to leave their child with a neighbor or other people to seek health care. They were concerned that their child would become infected with COVID-19 as a result of their inability to comply with the quarantine conditions in this situation.

*“When we have to go to the hospital in this situation, it is not just our pain; I am afraid of taking the virus and disease home with me. Once I become infected and return home, I’m afraid that both my husband and daughter will become infected too” (p 15).*

*“When we go to the clinic, I have to take my child to a neighbor’s house and that makes me so worried and scared because I don’t know if my neighbors are sick or not, if they are carriers or not, and if they are carriers, God forbid my child could get infected” (p 12).*

Another issue that caused women with high-risk pregnancies to experience negative feelings about receiving services was the issue of their transportation to receive health services, which comprised two aspects: the first was the fear and concern of becoming infected while using public transportation and the cost of personal vehicles, while the second was the concerns stemmed from traffic and road restrictions, particularly for mothers who lived far from facilities and had to travel to hospitals and equipped centers in big cities to receive health services. In this case, due to road closures and traffic bans, a woman with a high-risk pregnancy would experience tension, stress, confusion, and bewilderment. Furthermore, traffic restrictions beginning at 9 p.m. onwards had caused stress and anxiety in women with high-risk pregnancies who needed to visit for health services at that time.

*“There is no Doppler ultrasound in our city so I had to come here to have an ultrasound, but I was stressed out at that time cause the roads were closed because of Corona, and I was confused about what to do” (P 18).*

On the one hand, the fears, worries, and stresses of COVID-19, the need for quarantine and social distancing in the context of the COVID-19 pandemic, and on the other hand, the negative emotions perceived in high-risk pregnancies and the need for help and care from others had caused loneliness, sadness, and depression in the high-risk pregnant woman.

*“In this situation, the fear of Corona has added to my pregnancy problems. I can’t go anywhere, and no one can come to me. I’m lonely, I like to cry all the time, and I’m depressed. I need someone’s help because I can’t do any chores on my own”(P 1).*

##### Conflict and doubt

Women with high-risk pregnancies had to go to the hospital due to high-risk pregnancy conditions during the COVID − 19 pandemic, despite having negative feelings of developing COVID − 19 for their own and family members’ safety. As a result, they constantly experienced doubt and conflict in these situations.

On the other hand, some mothers admitted that the condition of a high-risk pregnancy was so distressing for them that when they went to the hospital, they forgot about the COVID-19 pandemic and no longer thought about it. In this regard, a mother with severe abdominal pain during pregnancy stated:

*“I was in so much pain at the time that I completely forgot about the Corona. I had no choice but to go to the hospital in those circumstances, even if I was scared to get Corona”(P 3).*

##### Feeling guilty

Guilt was another emotion that mothers with high-risk pregnancies experienced during the COVID-19 pandemic. They stated that they felt guilty for refusing to visit health centers or clinics for fear of developing COVID-19, especially when complications of high-risk pregnancies developed or worsened. They constantly blamed themselves for the harm done to the pregnancy, the fetus, or themselves. In this regard, the 33-year-old mother suffering from oligohydramnios during her third pregnancy states:

*“I feel very guilty about the lack of water around my baby now, and I can’t forgive myself at all. If I had come on time, there wouldn’t be so much water around my baby now, but I was afraid of getting Corona, so I didn’t come, and it’s like that now”(P 6).*

#### 2- adoption behavior

This category comprises three subcategories: “observing health protocols,” “Stop of Health Care”,” and “coping strategies to reduce negative emotions.” In the COVID-19 pandemic, this reflects the behavior of women with high-risk pregnancies.

##### Following health protocols

Many women with high-risk pregnancies stated that they had performed all the necessary care, tests, and ultrasounds to protect the fetus, despite the COVID-19 pandemic prevalence. Women tried to follow hygienic protocols such as using masks correctly, even wearing two masks, regular hand washing and disinfection with alcohol, wearing gloves, keeping a proper distance from others when receiving prenatal care, and washing and disinfecting clothing and utensils after receiving health services to prevent developing COVID-19.

*“Anyway, I did all the tests, ultrasounds, and care to keep the baby and my pregnancy safe. I did everything I could to avoid Corona. I was wearing mask and gloves, spraying my hands with alcohol all the time, and trying to keep my distance from others, so I wouldn’t get infected” (p 8).*

##### Refusal of health care

As described by mothers with high-risk pregnancies, they had refused to receive health services in some cases due to fear of developing Corona and social pressures such as intimidation from family and spouse on developing Corona, or lack of family’s consent even when hospitalization deemed necessary. Especially in the case of hospitalized mothers with high-risk pregnancies, even the health care staff would decide to leave the hospital as soon as the hospitalized patient started showing signs of illness or had positive COVID-19 test result.

*“As soon as I found out that a patient or a nurse had a positive test, I quickly gave my personal consent and left the hospital. My husband kept saying that in this situation, he does not want to go to the doctor permanently” (p 3).*

##### Coping strategies

Many women with high-risk pregnancies reported that thinking positively or not thinking about developing COVID-19 helped them adapt to the COVID-19 pandemic, reduce their fears, stresses, and worries, and gain control over their high-risk pregnancy conditions.

*“I try not to think negative thoughts at all; I generally believe that whatever I think happens to me. If I say what if I get Corona over and over, I’m going to get Corona”(P 12).*

Many mothers dealt with negative emotions by relying on spirituality and faith in God. They believed that death and life were in God’s hands and that God would protect her health and the fetus.

*“Now, when the doctor said you should be hospitalized in this hospital, I said, whatever God wants; and I was hospitalized” (p 3).*

#### 3- adjustment of health services in the mutual protection

This category was extracted from two subcategories, including supportive and preventive behavior of health care providers and access to health services. In the context of the COVID-19 pandemic, in-person and remote access to maternal health services for high-risk pregnancies reflected healthcare providers’ efforts to protect the health of staff and mothers at risk of COVID-19.

##### Supportive and protective behavior

During the COVID-19 pandemic, women with high-risk pregnancies described some of the healthcare providers’ behaviors as relaxing/comforting. Relaxing behaviors include Protective behaviors such as wearing two masks, gloves, and changing and disinfecting hands while caring for one patient to another, observing proper distance with patients when providing care, proper spacing of patients’ beds, preventing congestion when receiving health services, evaluating and testing for COVID-19 of all patients before hospitalization, and separating the hospital, ward, and rooms of patients infected with Corona from other patients. Health care provider’s supportive behaviors, such as providing appropriate and sufficient information to mothers and empathizing with mothers, helped them remain calm when receiving health services.

*“We feel calm and relaxed when we go to the hospital or clinic and see that the staff is wearing masks, gloves, and disinfecting their hands from patient to patient” (p 12).*

Some women with high-risk pregnancies described the lack of attention and support from health workers as stressful. Behaviors such as staff discussing the infection of pregnant women with coronavirus and reports of the death of a mother or fetus and her baby from COVID-19 in the presence of mothers with high-risk pregnancies increased the mother’s stress, anxiety, and fear, leading to her decision to leave the hospital.

Some women have acknowledged that healthcare providers’ care and attention have decreased in the wake of the COVID-19 pandemic. Some mothers, for example, reported receiving health care remotely, while staff refused to listen to fetal heart sounds or perform abdominal examinations that required close contact with the mother. During this time, some mothers with high-risk pregnancies reported shorter visits and staff’s refusal in taking the time to talk to mothers during visits or care. Although health workers’ behavior was intended to protect against Corona, it caused women with high-risk pregnancies to feel emotions such as stress, anxiety, and neglect while receiving health services.

*“I feel like the staff is less caring and they don’t take as much time caring as they used to. The pregnancy care is very short without any talks, well, they have to be careful not to get Corona themselves” (P 22).*

##### Access to health services

Some mothers reported that during the initial announcement of the COVID-19 pandemic, some doctors closed their offices, causing them to stress and confusion. Due to transportation restrictions during COVID-19 pandemic, some high-risk pregnant women complained of drug shortages.

“I have a heart condition. After we were told about the Corona, the doctor’s offices closed right away. My cardiologist also closed his office. I was worried because I couldn’t find my warfarin medication anywhere; they said it didn’t enter because the transport system was closed.”

Despite the COVID-19 pandemic, many mothers with high-risk pregnancies reported having access to health care, either in person or remotely. Some even stated that due to the lack of crowd congestion during the COVID-19 outbreak, access to health care facilities became easier. They stated that it was possible to call a doctor or midwife 24 h a day. During the COVID-19 pandemic, it was also possible to send a picture of the tests and ultrasound to the doctor and midwife and receive their opinion.

*“When I felt I had a problem, I called or texted the midwife at the health center at any time of day or night; they always answered and often said it’s not necessary to bring the test or the ultrasound results, sending the pictures is enough”(p 18).*

## Discussion

In the COVID-19 pandemic condition, women with high-risk pregnancies experience negative psychological responses such as fear, anxiety, and worry, guilt, doubt, confusion, and conflict. They inevitably receive health care, followed by feelings of depression, sadness, and loneliness. In these cases, women adopted thought-based behaviors to protect themselves and their pregnancies from the double risks of the COVID-19 and high-risk pregnancies. Positive effects of the healthcare system’s behavior and environment, on the other hand, were beneficial in converting those negative emotions and behaviors into positive emotions and health-promoting behaviors. As a result of receiving appropriate information, protective behaviors, and empathetic care from healthcare providers, women’s negative emotions are hopefully reduced and replaced by a sense of tranquility and confidence.

This qualitative study contributes to the scientific understanding of how women with high-risk pregnancies experience increased emotional distress in the context of COVID-19. On the one hand, they are distressed by a high-risk pregnancy, while on the other, they are concerned that they or their families may become infected with COVID-19 and suffer the limitations that this entails. Guilt was a recurring emotion for them because they held themselves responsible for their own and their fetus’s health during COVID-19. Consequently, they avoided receiving health services to reduce their risk of developing COVID-19 and comply with the social transit distance; however, when high-risk pregnancy complications occurred, they felt guilty for failing to visit on time. This study also shows that the COVID-19 pandemic has an impact on the health behaviors of mothers with high-risk pregnancies. Some mothers refused prenatal care out of fear of illness and social pressures, while others tried to avoid delaying health care by adhering to health protocols. Many mothers in these situations sought medical attention and managed their emotional stress by employing coping strategies such as positive thinking and faith in God. Furthermore, the study’s findings suggest that, in addition to protecting health care workers from COVID-19, prevention behaviors by health care workers provide comfort to mothers with high-risk pregnancies. In addition, the presence of COVID-19 protective strategies in health care settings reduced the stress of mothers with high-risk pregnancies. Health care providers’ supportive and empathetic behaviors, such as providing appropriate and correct information to mothers with high-risk pregnancies, make them feel good about receiving health services during the COVID-19 pandemic. No matter the conditions of COVID-19, receiving health services, whether in person, by phone, or online, created a feeling of positive wellbeing in mothers with high-risk pregnancies.

Regarding negative psychological responses, Kotabagi writes: “Psychological disorders have doubled during the Corona outbreak. The mortality, high prevalence, and the nature of the COVID-19 pandemic have increased fears, stress, and anxiety globally. Social isolation and its consequences, on the other hand, are significant factors in psychological disorders” [34, 35]. Also, in high-risk pregnancies, women increasingly feel that due to not receiving adequate care, something life-threatening and harmful may occur to the fetus, which is the psychological outcome of the COVID-19 pandemic [25].

Evidence suggests that quarantine and social isolation have negative psychological effects on women with high-risk pregnancies, including symptoms of post-traumatic stress disorder, confusion, and anger. According to Katabatic (2020) the average score of anxiety and depression among British pregnant women rises with the peak of COVID-19 mortality and uncertainty about healthcare capacity to control COVID-19. In contrast, receiving useful information from health care providers reduces negative emotions in women with high-risk pregnancies during the COVID-19 pandemic [34, 36]. In any case, the mental health of women with high-risk pregnancies during the COVID-19 pandemic should not be overlooked [34]. Negative psychological responses during pregnancy increase the risk of preeclampsia, preterm delivery, and low birth weight [37], and also anxiety in pregnant mothers can increase labor pain [38] while providing timely psychological support to high-risk pregnant women during COVID-19 pandemic can be very effective in preventing such negative consequences. Psychological support for mothers with high-risk pregnancies in the face of the Corona pandemic condition requires a special investment by the government, as it has the potential to benefit society greatly in the future [23, 39]. According to Lebela, social support can alleviate the psychological pressures and concerns caused by COVID-19 [25].

The study’s findings point to the effects of the COVID-19 pandemic on health-seeking behaviors of women with high-risk pregnancies, as well as the adoption of thought-based behaviors to protect against the double danger of COVID-19 in high-risk pregnancies. In fact, pregnant women’s fear of developing COVID-19 reduced the number of referrals for prenatal care, which was associated with feelings of guilt or doubt. According to the studies, teleconsultation, sometimes referred to as remote consultation or telehealth, refers to providing counseling and health services over the phone, video conferencing, or the Internet, has been shown to improve depression, anxiety, quality of life, and psychosocial functioning, as well as the quality of pregnancy care among pregnant women during the Corona pandemic condition [40]. Thus, during the COVID-19 pandemic, it is recommended that women with high-risk pregnancies use technology for prenatal care and psychological support [25]. Also, husband support and peer-assisted training programs can significantly improve antenatal care and physical and mental disorders in women with high-risk pregnancies in areas with limited access to technology [41, 42]. Receiving at least four phone calls per day from trained peers or health care providers to ensure the pregnant mother’s health status will reduce depressive symptoms in pregnant women [43].

Similar to the findings of the current study, in another study conducted in New York City, Aleha Aziz (2020) found that many mothers with high-risk pregnancies considered commuting and using vehicles to be a problem. In addition to worrying about the risk of using public transportation, they also complained about the cost of using personal vehicles [44, 45]. Therefore, to help solve this problem, they suggested using telehealth. They believe that, in addition to reducing the risk of COVID-19, telehealth comes with social and economic advantages in caring for mothers with high-risk pregnancies. This method is an excellent replacement for face-to-face visits in prenatal care and high-risk pregnancies. To ensure adequate access to health services, they recommend adopting a combination of face-to-face and telehealth care methods [44]. Some researchers, including Rhodes, have proposed using a digital program to replace face-to-face health services in the United Kingdom during the COVID-19 pandemic to improve the quality of prenatal care for women with high-risk pregnancies [46]. According to the results of the present study, many mothers adapted to the COVID-19 pandemic condition by adhering to the health protocols and preventive behaviors, as well as taking coping strategies such as positive thinking and trust in God while performing prenatal care to maintain their health and that of the fetus. According to research, coping strategies, reliance on spirituality, and risk-control behaviors during pregnancy are all prerequisites for well-being in high risk pregnancy [47]. The findings of the Koenig (2020) study also illustrate the importance of faith in mitigating the effects of the disease, even in COVID-19 [48].

According to the findings of the study, health care providers’ behaviors and environment play an important role in the optimal reception of health services during the COVID-19 pandemic. The behaviors aimed at modifying health care services with reciprocal care for staff and pregnant women to prevent COVID-19. A review of the literature reveals that during pandemics, many midwives and nurses continue to practice with passion, interest, and diligence, providing empathetic and supportive care to patients. They strive to provide the best and highest quality care possible. And, in the event of a disease epidemic, provide a suitable environment for the provision of health services to prevent disease [40, 49]. Because they believe that gaining knowledge and experience is rewarding, and pandemic services allow them to gain clinical experience, confidence, and competence. It also fosters trust and value in patients, their families, and society as a whole. They have a sense of purpose and meaning in their work. Their goal is to provide quality services with the least harm to clients and recipients of health services. In such cases, midwives and nurses use their competencies and creativity to develop better solutions for providing health services to patients cope with health system failures [49, 50]. Seeing such behaviors in the healthcare delivery system gives women with high-risk pregnancies confidence in receiving healthcare services. The US Food and Drug Administration (2020) also emphasizes the critical role of health care professionals during the COVID-19 pandemic [51].

### Research strengths, limitations, and future research suggestions

The strength of the current study is conducting qualitative research through in-depth interviews with high-risk pregnant women, allowing them to learn about their perceptions and experiences while revealing potential barriers and factors that facilitate obtaining optimal pregnancy care in the context of the COVID-19 pandemic. Many of these aspects are overlooked in quantitative studies. However, the present study has also faced some limitations, including the need to exercise caution when generalizing the findings, which is a limitation of all qualitative research. Another limitation was that only women with high-risk pregnancies had an in-depth interview to extract the findings of receiving prenatal care. As a result, conducting qualitative studies with health care providers is recommended in this field. Also, a study should be conducted to compare prenatal care receipt in low-risk and high-risk pregnancies in the context of the COVID-19 pandemic.

## Conclusion

Receiving pregnancy care in women with high-risk pregnancies can impose challenges on women in the COVID-19 pandemic that can adversely affect their psychological reactions and health-seeking behaviors. They experience both the emotional distress of a high-risk pregnancy and the distress of developing COVID-19 in themselves and their families while in health care. By following health and safety protocols, women with high-risk pregnancies can maintain their health-seeking behaviors; however, in some cases, they may refuse to seek health services. Preventive care against COVID-19 by health care workers will not only protect them from infection but will also create a positive well-being perception to encourage women with high-risk pregnancies to receive health services during the COVID-19 pandemic. Receiving empathetic and supportive behavior from health care providers makes them feel at ease and well-received, which can both ensure control of high-risk pregnancies and protect women with high-risk pregnancies from the risk of developing COVID-19. Women can feel better by implementing coping strategies and having access to health care. As a result, policymaking, planning, and investing in the care of high-risk pregnant women should focus on preventing COVID-19 while minimizing pregnancy risks and complications. Research findings support clinical and educational services, so while planning, policy-making, and investing in the care of mothers with high-risk pregnancies, mothers’ psychological distress should be focused on while providing them with psychological screenings and counseling. The health care facilities should be able to protect patients from COVID-19. Staff must follow health and safety protocols and provide adequate training to mothers with high-risk pregnancies to prevent COVID-19 infections. Women should also receive care with empathy and in a woman-centered manner. Access to remote health services and technology-based care is recommended during the COVID-19 pandemic to reduce pregnancy risks and complications.

## Data Availability

Although we do not have consent from all patients to publish this data, we keep the names and information of all participants confidential. As a result, the data supporting the findings of this study are available upon reasonable request from the corresponding author (Tkhadivzadeh@mums.ac.ir) without jeopardizing participant confidentiality.

## References

[1] Cucinotta D, Vanelli M (2020). WHO declares COVID-19 a pandemic. Acta Bio Medica.

[2] Abdi M (2020). Coronavirus disease 2019 (COVID-19) outbreak in Iran: Actions and problems. Infect Control Hosp Epidemiol.

[3] Asadi S, Bouvier N, Wexler AS, Ristenpart WD (2020). The coronavirus pandemic and aerosols: Does COVID-19 transmit via expiratory particles?.

[4] Zhou F, Yu T, Du R, Fan G, Liu Y, Liu Z (2020). Clinical course and risk factors for mortality of adult inpatients with COVID-19 in Wuhan, China: a retrospective cohort study. Lancet.

[5] Francesca Donders M, Lonnee-Hoffmann R, Mendling M, de Oliveira JM, Judlin P, Fengxia X (2020). ISIDOG recommendations concerning COVID-19 and pregnancy. Diagnostics (Basel).

[6] Lam CM, Wong SF, Leung TN, Chow KM, Yu WC, Wong TY (2004). A case-controlled study comparing clinical course and outcomes of pregnant and non-pregnant women with severe acute respiratory syndrome. BJOG.

[7] Schwartz DA (2020). An analysis of 38 pregnant women with COVID-19, their newborn infants, and maternal-fetal transmission of SARS-CoV-2: maternal coronavirus infections and pregnancy outcomes. Arch Pathol Lab Med.

[8] Van Otterloo LR, Connelly CD (2016). Maternal risk during pregnancy: a concept analysis. J Clin Nurs.

[9] Michel KN, Ilunga BC, Astrid KM, Blaise IK, Mariette KK, Pitchou KT (2016). Epidemiological profile of high-risk pregnancies in Lubumbashi: case of the provincial hospital Janson Sendwe. Open Access Lib J.

[10] Bajalan Z, Sabzevariha Z, Abdollahi F, Qolizadeh A (2019). Prevalence of High-risk Pregnancies and the Correlation between the Method of Delivery and the Maternal and Neonatal Outcomes. J Pediatr Nurs.

[11] Mirzadeh M, Khedmat L (2020). Pregnant women in the exposure to COVID-19 infection outbreak: the unseen risk factors and preventive healthcare patterns. J Matern Fetal Neonatal Med.

[12] Dong M, Qian R, Wang J, Fan J, Ye Y, Zhou H (2021). Associations of COVID-19 Lockdown with Gestational Length and Preterm Birth in China. BMC.

[13] Palmrich P, Roessler B, Wisgrill L, Kampf S, Gattinger P, Valenta R (2021). Multiprofessional perinatal care in a pregnant patient with acute respiratory distress syndrome due to COVID-19. BMC Pregnancy Childbirth.

[14] NeJhaddadgar N, Ziapour A, Zakkipour G, Abbas J, Abolfathi M, Shabani M (2022). Effectiveness of telephone-based screening and triage during COVID-19 outbreak in the promoted primary healthcare system: a case study in Ardabil province, Iran. J Public Health..

[15] Norooznezhad AH, Nurzadeh M, Darabi MH, Naemi M (2021). Coronavirus disease 2019 (COVID-19) in a pregnant women with treatment resistance thrombocytopenic purpura with and suspicion to HELLP syndrome: a case report. BMC Pregnancy Childbirth.

[16] Ranjbar F, Allahqoli L, Ahmadi S, Mousavi R, Gharacheh M, Eshraghi N (2021). Changes in pregnancy outcomes during the COVID-19 lockdown in Iran. BMC Pregnancy Childbirth.

[17] Chen J, Cai Y, Liu Y, Qian J, Ling Q, Zhang W (2016). Factors associated with significant anxiety and depressive symptoms in pregnant women with a history of complications. Shanghai Arch Psychiatr.

[18] Roberts RM, Muller T, Sweeney A, Bratkovic D, Gannoni A (2014). Promoting psychological well-being in women with phenylketonuria: pregnancy-related stresses, coping strategies and supports. Mol Genet Metab Rep.

[19] Yassa M, Birol P, Yirmibes C, Usta C, Haydar A, Yassa A (2020). Near-term pregnant women’s attitude toward, concern about and knowledge of the COVID-19 pandemic. J Matern Fetal Neonatal Med.

[20] Wang D, Hu B, Hu C, Zhu F, Liu X, Zhang J (2020). Clinical characteristics of 138 hospitalized patients with 2019 novel coronavirus–infected pneumonia in Wuhan, China. Jama.

[21] Hantsoo L, Kornfield S, Anguera MC, Epperson CN (2019). Inflammation: a proposed intermediary between maternal stress and offspring neuropsychiatric risk. Biol Psychiatr.

[22] Özdin S, Bayrak ÖŞ (2020). Levels and predictors of anxiety, depression and health anxiety during COVID-19 pandemic in Turkish society: The importance of gender. Int J Soc Psychiatr.

[23] Abbas J (2020). The impact of coronavirus (SARS-CoV2) epidemic on individuals mental health: the protective measures of Pakistan in managing and sustaining transmissible disease. Psychiatria Danubina.

[24] Wang C, Wang D, Abbas J, Duan K, Mubeen R (2021). Global financial crisis, smart lockdown strategies, and the COVID-19 spillover impacts: A global perspective implications from Southeast Asia. Front Psychiatr.

[25] Lebel C, MacKinnon A, Bagshawe M, Tomfohr-Madsen L, Giesbrecht G (2020). Elevated depression and anxiety symptoms among pregnant individuals during the COVID-19 pandemic. J Affect Disord.

[26] Peahl AF, Smith RD, Moniz MH (2020). Prenatal care redesign: creating flexible maternity care models through virtual care. Am J Obstet Gynecol.

[27] Holloway I, Galvin K (2016). Qualitative research in nursing and healthcare.

[28] Shoorab NJ, Taghipour A, Esmaily H, Roudsari RL (2020). Development and Psychometric Properties of the Women’s Recovery of Postnatal Perineal Injuries Questionnaire (WRPPIQ). Int J Community Based Nurs Midwifery.

[29] Bengtsson M (2016). How to plan and perform a qualitative study using content analysis. Nurs Plus Open.

[30] Tenny S, Brannan GD, Brannan JM, Sharts-Hopko NC. Qualitative study. 2017. https://europepmc.org/article/NBK/nbk470395#free-full-text.

[31] Holness N (2018). High-risk pregnancy. Nurs Clin.

[32] Graneheim UH, Lundman B (2004). Qualitative content analysis in nursing research: concepts, procedures and measures to achieve trustworthiness. Nurs Educ Today.

[33] Lincoln YS, Guba EG (1985). Naturalistic inquiry.

[34] Kotabagi P, Fortune L, Essien S, Nauta M, Yoong W (2020). Anxiety and depression levels among pregnant women with COVID-19. Acta Obstetricia Et Gynecologica Scandinavica.

[35] Shuja KH, Aqeel M, Khan EA, Abbas J (2020). Letter to highlight the effects of isolation on elderly during COVID-19 outbreak. Int J Geriatr Psychiatr.

[36] Su Z, McDonnell D, Wen J, Kozak M, Abbas J, Šegalo S (2021). Mental health consequences of COVID-19 media coverage: the need for effective crisis communication practices. Global Health.

[37] Hasanjanzadeh P, Faramarzi M (2017). Relationship between maternal general and specific-pregnancy stress, anxiety, and depression symptoms and pregnancy outcome. JCDR.

[38] Shourab NJ, Zagami SE, Golmakhani N, Mazlom SR, Nahvi A, Pabarja F (2016). Virtual reality and anxiety in primiparous women during episiotomy repair. Iran J Nurs Midwifery Res.

[39] Lebel C, MacKinnon A, Bagshawe M, Tomfohr-Madsen L, Giesbrecht G (2021). Corrigendum to elevated depression and anxiety symptoms among pregnant individuals during the COVID-19 pandemic journal of affective disorders 277 (2020) 5-13. J Affect Disord.

[40] Shorey S, Valerie C (2020). Lessons from past epidemics and pandemics and a way forward for pregnant women, midwives and nurses during COVID-19 and beyond: A meta-synthesis. Midwifery.

[41] Pfeiffer B, Kramer J, Roth S. Peer Support and Intellectual or Developmental Disability (IDD) Rapid Review Search. Strategy. 2020;12:21–47. 10.34944/dspace/4645.

[42] Kobra Mirzakhani TK, Faridhosseini F, Ebadi A (2020). Pregnant women’s experiences of the conditions affecting marital well-being in high-risk pregnancy: A qualitative study. Int J Community Based Nurs Midwifery.

[43] Dennis C-L, Hodnett E, Kenton L, Weston J, Zupancic J, Stewart DE (2009). Effect of peer support on prevention of postnatal depression among high risk women: multisite randomised controlled trial. Bmj.

[44] Aziz A, Zork N, Aubey JJ, Baptiste CD, D'alton ME, Emeruwa UN (2020). Telehealth for high-risk pregnancies in the setting of the COVID-19 pandemic. Am J Perinatol.

[45] Abbas J, Mubeen R, Iorember PT, Raza S, Mamirkulova G (2021). Exploring the impact of COVID-19 on tourism: transformational potential and implications for a sustainable recovery of the travel and leisure industry. Curr Res Behav Sci.

[46] Rhodes A, Kheireddine S, Smith AD (2020). Experiences, Attitudes, and Needs of Users of a Pregnancy and Parenting App (Baby Buddy) During the COVID-19 Pandemic: Mixed Methods Study. JMIR mHealth uHealth.

[47] Mirzakhani K, Ebadi A, Faridhosseini F, Khadivzadeh T (2020). Well-being in high-risk pregnancy: an integrative review. BMC Pregnancy Childbirth.

[48] Koenig HG (2020). Maintaining health and well-being by putting faith into action during the COVID-19 pandemic. J Relig Health.

[49] Erland E, Dahl B (2017). Midwives’ experiences of caring for pregnant women admitted to Ebola centres in Sierra Leone. Midwifery.

[50] Kollie ES, Winslow BJ, Pothier P, Gaede D (2017). Deciding to work during the Ebola outbreak: the voices and experiences of nurses and midwives in Liberia. Int J Afr Nurs Sci.

[51] Risko N, Werner K, Offorjebe OA, Vecino-Ortiz AI, Wallis LA, Razzak J (2020). Cost-effectiveness and return on investment of protecting health workers in low-and middle-income countries during the COVID-19 pandemic. PloS One.

